# Chronic kidney disease and use of dental services in a united states public healthcare system: a retrospective cohort study

**DOI:** 10.1186/1471-2369-13-16

**Published:** 2012-04-02

**Authors:** Vanessa Grubbs, Laura C Plantinga, Delphine S Tuot, Neil R Powe

**Affiliations:** 1 San Francisco General Hospital, Division of Nephrology, University of California, San Francisco, USA; 2 Department of Epidemiology, Emory University Rollins School of Public Health, Atlanta, USA; 3 San Francisco General Hospital, Department of Medicine, University of California, San Francisco, USA

## Abstract

**Background:**

As several studies have shown an association between periodontal disease and chronic kidney disease (CKD), regular dental care may be an important strategy for reducing the burden of CKD. Access to dental care may be limited in the US public health system.

**Methods:**

In this retrospective cohort study of 6,498 adult patients with (n = 2,235) and without (n = 4,263) CKD and at least 12 months of follow-up within the San Francisco Department of Public Health Community Health Network clinical databases, we examined the likelihood of having a dental visit within the observation period (2005-2010) using Cox proportional hazards models. To determine whether dental visits reflected a uniform approach to preventive service use in this setting, we similarly examined the likelihood of having an eye visit among those with diabetes, for whom regular retinopathy screening is recommended. We defined CKD status by average estimated glomerular filtration rate based on two or more creatinine measurements ≥ 3 months apart (no CKD, ≥ 60 ml/min/1.73 m^2^; CKD, < 60 ml/min/1.73 m^2^).

**Results:**

Only 11.0% and 17.4% of patients with and without CKD, respectively, had at least one dental visit. Those with CKD had a 25% lower likelihood of having a dental visit [HR = 0.75, 95% CI (0.64-0.88)] than those without CKD after adjustment for confounders. Among the subgroup of patients with diabetes, 11.8% vs. 17.2% of those with and without CKD had a dental visit, while 58.8% vs. 57.8% had an eye visit.

**Conclusions:**

Dental visits, but not eye visits, in a US public healthcare setting are extremely low, particularly among patients with CKD. Given the emerging association between oral health and CKD, addressing factors that impede dental access may be important for reducing the disparate burden of CKD in this population.

## Background

Chronic kidney disease (CKD) affects an estimated 14% of the adult US population [1]. Over a half million adults have progressed to end-stage renal disease, which confers considerable societal cost—in excess of $30 billion in 2008 [2]—and is the ninth leading cause of death in the United States [3]. Further, CKD disproportionately affects racial/ethnic minorities and the poor [4-8]—populations that are over-represented in public healthcare systems [9,10]. Prevention of CKD progression is key for reducing racial-ethnic and socioeconomic disparities and societal burden of CKD.

Regular dental care may be an important strategy for preventing CKD progression, given that several studies have shown individuals with significant periodontal disease have 1.5- to 2-fold increased odds of CKD, even after accounting for important confounders such as diabetes, tobacco use, and socioeconomic status [11-14]. Though periodontal disease is a local bacterial infection of the oral cavity, periodontal pathogens can access systemic circulation [15,16] and potentially induce kidney injury through an innate immune response. However, in the resource-poor public healthcare setting, recommended regular preventive dental care may not be readily accessible. Further, patients with CKD may be less apt to seek dental care because of excess appointments and lifestyle modifications required for CKD management.

To help understand use of dental services within a public healthcare setting, we described dental visits among adults with and without CKD receiving care in the San Francisco Department of Public Health Community Health Network. To determine whether use of dental services reflected a uniform approach to preventive service use in this population, we also described eye visits among the subgroup with diabetes (a strong risk factor for both CKD [17] and periodontal disease [18])-- for whom retinopathy screening is recommended in standard guidelines for diabetes care [19].

## Methods

### Study design and population

The Health Record Data Service (THREDS) provides longitudinal data from electronic clinical databases (Lifetime Clinical Record and Invision) within the San Francisco Department of Public Health Community Health Network for research purposes. The Network is an integrated public healthcare system that includes federally qualified health centers and shares an electronic medical record system with unique patient identifiers to support clinical and research activities across sites. We requested that THREDS provide randomly selected cohorts starting in January 1, 2005 of records from 5,000 adults (≥ 20 years) with CKD, 5,000 without CKD, and 5,000 of unknown CKD status. For this study, we included those adults with and without CKD who had at least two primary care visits on or after January 1, 2005; and at least 12 months of follow-up before July 31, 2010. This study was approved by the University of California, San Francisco Committee on Human Research.

### Measurements and definitions

We defined CKD by two outpatient serum creatinine measurements at least 3 months apart associated with an average estimated glomerular filtration rate (eGFR) < 60 ml/min/1.73 m^2^. No CKD was defined by no creatinine values associated with eGFR < 60 ml/min/1.73 m^2^, or by one abnormal creatinine value followed by a normal value. Unknown CKD status was defined by zero or only one creatinine measurement. Estimated GFR was calculated according to the Modification of Diet in Renal Disease (MDRD) Study equation for calibrated creatinine [20], as is used clinically in the study population setting for detection of CKD. Serum creatinine was measured using an ADVIA chemistry analyzer (Siemens Healthcare Diagnostics, Deerfield, IL) by the Jaffe method before June 2008 and by enzymatic assay after June 2008.

A dental visit was defined as an encounter at any Network dental facility (inpatient or outpatient) at any time during the study period, which included the time from study entry (date of second creatinine confirming CKD status) to last date of follow-up (July 31, 2010) or death. Similarly, we defined an eye visit as an encounter at any Network eye facility (inpatient or outpatient) at any time during the study period. Diabetes was defined by any International Classification of Diseases, 9^th ^Revision, Clinical Modification (ICD-9-CM) diagnostic code in the medical record for diabetes (250.xx) during the study period. Periodontal disease was defined by any ICD-9-CM diagnostic code in the medical record for periodontal disease (523.xx) or loss of teeth due to periodontal disease (525.12).

Covariates were defined at study entry. We categorized self-reported age into three groups (20-45, 46-64, 65+) and self-reported race/ethnicity as white, black, Latino, Asian, or other. Language preference was categorized as English, Spanish, Chinese (Cantonese or Mandarin), or other. Insurance status was categorized as Medicare, Medicaid, or other (city plan, county assistance, self-pay, or worker's compensation). Monthly income was categorized as $0, $1-$500, or $501-$1,000.

### Statistical analyses

Patient characteristics were examined by CKD status using chi-square analyses. Fewer than 10% of the cohort had more than 1 dental visit during the study period. Therefore, we fit Cox proportional hazards models to examine likelihood of having a dental visit by CKD status at any time during the study period after study entry. Covariates were added to the unadjusted model in a sequential fashion with the final model including age group, gender, race/ethnicity, language preference, insurance, and monthly income.

Additionally, we examined having any dental visit or any eye visit by CKD status among patients with and without ICD-9 code for diabetes using chi-square analyses. We repeated Cox proportional hazard models as above to separately test the association between time to any dental visit or any eye visit and CKD by diabetes status. All analyses were performed with Stata v. 12.0 (StataCorp, College Station, TX).

## Results

We included 2,235 and 4,263 adult (> = 20 years) patients with and without CKD, respectively, who met our study criteria. Patient characteristics by CKD status are shown in Table 1. Patients in the CKD cohort were more often over age 65, of Asian race/ethnicity, had diabetes, and insured by Medicare than those without CKD.

**Table 1:** Patient characteristics by CKD status

Characteristic	No CKD n = 4,263	CKD n = 2,235	P-value
Had dental visit, n (%)	741 (17.4)	245 (11.0)	< 0.001

Years follow-up, mean (SD)	4.3 (1.2)	4.5 (1.2)	< 0.001

Age group, n (% characteristic)			< 0.001

20-45	1,210 (28.4)	215 (9.6)	

46-64	2,250 (52.8)	1,011 (45.2)	

65+	803 (18.8)	1,009 (45.1)	

Gender, n (% characteristic)			0.001

Male	2,193 (51.4)	1,052 (47.1)	

Female	2,070 (48.6)	1,182 (52.9)	

Race/Ethnicity, n (% characteristic)			< 0.001

White	996 (23.5)	490 (22.1)	

Black	1,283 (30.3)	500 (22.6)	

Hispanic	893 (21.1)	382 (17.2)	

Asian	986 (23.3)	781 (35.2)	

Other	80 (1.9)	64 (2.9)	

Language, n (% characteristic)			< 0.001

English	2,958 (70.4)	1,467 (67.0)	

Spanish	568 (13.5)	234 (10.7)	

Chinese	443 (10.6)	306 (14.0)	

Other	232 (5.5)	182 (8.3)	

Diabetes			< 0.001

No	2,871 (67.3)	1,052 (47.1)	

Yes	1,392 (32.7)	1,183 (52.9)	

Insurance, n (% characteristic)			< 0.001

Medicare	1,381 (32.4)	1,275 (57.1)	

Medicaid	1,226 (28.8)	576 (25.8)	

Other	1,654 (38.8)	382 (17.1)	

Monthly income			< 0.001

$0	1,415 (33.2)	649 (29.0)	

$1-$500	1,780 (41.8)	1,060 (47.4)	

$501-$1000	1,068 (25.0)	526 (23.5)	

Although there was a wide range in total number of dental visits (range 0-46), only 986 (15.2%) of the entire study population had at least one dental visit over the observed period. Patients with CKD had a slightly longer mean follow-up period than those without CKD (4.5 vs. 4.3 years, p < 0.001), but less frequently had a dental visit (11.0% vs. 17.4%, p < 0.001). Among patients who had a dental visit, 33.8% and 32.9%, respectively, were diagnosed with periodontal disease (p = 0.767).

Adult patients with at least 12 months of follow-up but unknown CKD status (and therefore not included in the analysis, n = 3,275) were mostly under age 45 (56.7%), uninsured (65.8%), and did not have diabetes (90.8%). This group had a shorter mean follow-up period than those of known CKD status (3.8 vs. 4.4 years, p < 0.001) but a similar percentage had a dental visit (14.5% vs. 15.2%, p = 0.403) as those of known CKD status.

In the Cox proportional hazards model, those with CKD had a 27% lower likelihood of having a dental visit after adjusting for age [hazard ratio 0.73, 95% CI (0.63, 0.85)]. This association was similar after additionally adjusting for gender, race/ethnicity, language preference, insurance, and monthly income [0.75 (0.64-0.88)] (Table 2).

**Table 2:** Hazard ratios for having a dental visit by CKD status among entire cohort (reference = no CKD)

	Hazard Ratio	95% CI	P-value
unadjusted	0.60	0.52, 0.69	< 0.001

+ age	0.73	0.63, 0.85	< 0.001

+ gender, race/ethnicity, language	0.76	0.65, 0.88	< 0.001

+ insurance, monthly income	0.75	0.64, 0.88	< 0.001

Of note, in the fully adjusted model, the likelihood of having a dental visit significantly differed by age and race/ethnicity, but not insurance (data not shown). Age 46-64 and age over 65 were associated with a 38% [0.72 (0.62-0.83)] and 54% [0.46 (0.37-0.58)], respectively, lower likelihood of having a dental visit compared to age 20-45. Blacks were nearly twice as likely to have a dental visit as whites [1.88 (1.57-2.25)], but there was no difference in likelihood in having a dental visit between whites and other racial/ethnic groups. Tests for interaction between CKD status and age group or race/ethnicity were not significant (p_interaction _= 0.281 and 0.091, respectively).

**Figure 1:**
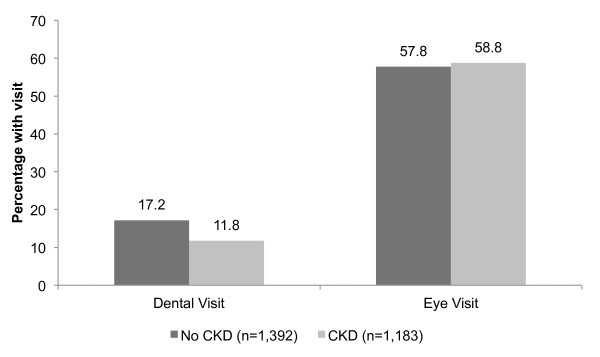
**Percentage of patients with diabetes who had at least one dental or eye visit during the study period, by CKD status**.

**Figure 2:**
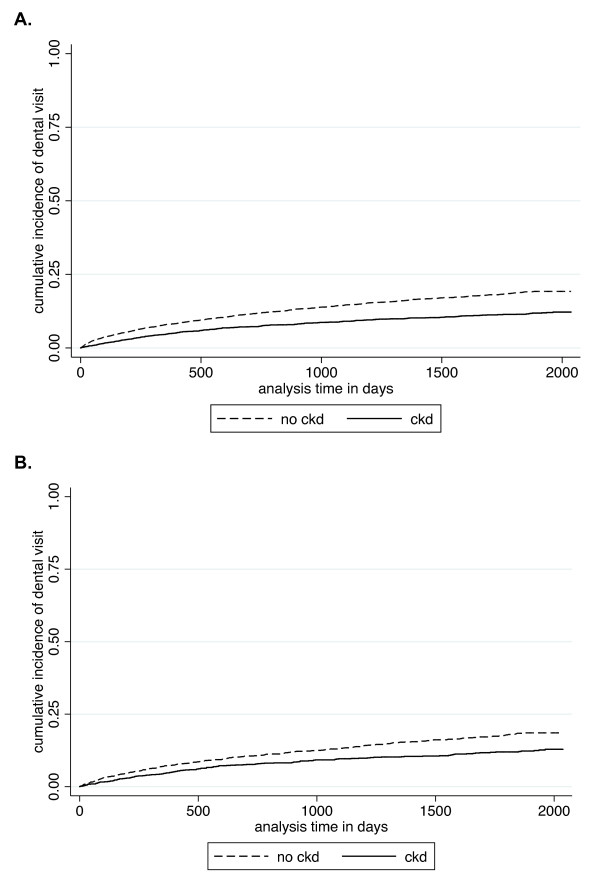
**Cumulative incidence of time to dental visit (A) overall and (B) among those with diabetes**.

**Figure 3:**
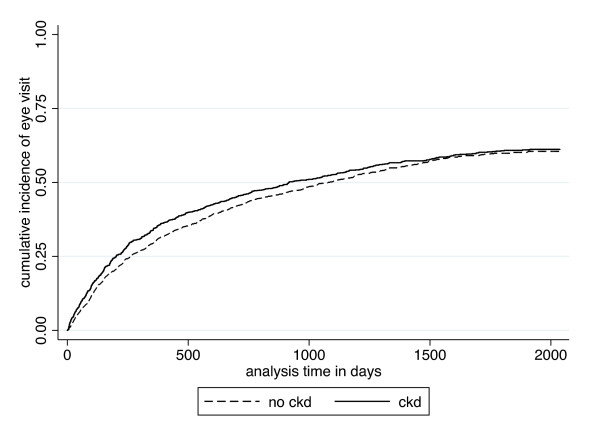
**Cumulative incidence of time to eye visit among those with diabetes**.

While the percentage of patients with a dental visit was similar between those with and without diabetes (14.7% vs. 15.5%, p = 0.368), significantly more patients with diabetes had an eye visit than those without diabetes (58.3% vs. 26.8, p < 0.001). The percentages of patients with diabetes with a dental or eye visit by CKD status are shown in Figure 1. Among the 1,392 patients with diabetes but no CKD, 239 (17.2%) had a dental visit while 805 (57.8%) had an eye visit over the entire study period. Among the 1,183 patients with diabetes and CKD, 139 (11.8%) had a dental visit and 696 (58.8%) had an eye visit. Similar to the entire study population, patients with diabetes and CKD had a 20% lower likelihood of having a dental visit than those without CKD in the fully adjusted Cox model [0.80, (0.64-1.00)]. However, there was no difference in likelihood of having an eye visit by CKD status [fully adjusted model, 1.07, (0.96-1.19)]. As depicted in Figure 2, the cumulative incidence of time to dental visit by CKD status is not substantially different in the entire study population versus the population restricted to those with diabetes by CKD status. Figure 3 depicts an essentially overlapping cumulative incidence by CKD status of time to eye visit among the subpopulation with diabetes.

## Discussion

This is the first study, to our knowledge, to examine dental visits by CKD status. Among a population of adult patients in a public healthcare setting in urban California, we found that the percentage of patients who had at least one dental visit during an average of > 4 years of observation was extremely low overall and that those with CKD had a 25% lower likelihood of having a dental visit than those without CKD. We found a similar difference in likelihood of having a dental visit by CKD status in the subgroup with diabetes, but no difference in the likelihood of having an eye visit by CKD status. Further, while the percentage having a dental visit was not significantly different between subgroups with or without diabetes, more than twice as many patients with diabetes than without diabetes had an eye visit.

These findings are important because of the emerging association between oral health and CKD. Though there have been no longitudinal studies to our knowledge examining the association of periodontal disease with incident or progressive CKD, cross-sectional associations have been reported [11-14] and its plausibility is supported in the cardiovascular literature where periodontal disease has been shown to be an independent risk factor for incident coronary heart disease [21], periodontal pathogens have been shown to be able to directly invade human coronary artery endothelial cells [22], and there is evidence of reversibility of effects on endothelial function [23]. One proposed mechanism for how periodontal pathogens may induce kidney injury is through toll like receptor 4 (TLR4)--which is one in a group of transmembrane proteins that play a key role in the innate immune response. TLR4 is found throughout the kidney [24,25] where it can recognize and bind bacterial lipopolysaccharide coating, and launch an inflammatory cascade that could lead to renal dysfunction [24,25]. Because periodontal disease may contribute to the development and progression of CKD and because it is preventable and treatable with regular dental care, a paucity of dental care in the public healthcare setting may be contributing to the disparate burden of CKD among the poor and racial/ethnic minorities. Our finding that a periodontal disease diagnosis was common among those with a dental visit lends support for the scope of its possible past and future impact.

Several factors may explain our findings. A lack of public insurance coverage of dental services undoubtedly creates a significant barrier to dental care access among the poor. Those with dental insurance or the ability to pay out-of-pocket are significantly more likely to utilize dental services than those without such resources [26,27]. Roughly 1 in 3 adults in the United States lacks dental insurance [28]. A lack of dental insurance is higher among the poor and medically uninsured [26,29]. Though the majority of our study population had Medicare (a federal health insurance program for adults over age 65 and those who are physically disabled or meet other special criteria) or Medicaid (a state-managed health insurance program for low income citizens and those with special disabilities), Medicare does not cover basic or routine dental care [30] and provision of adult dental services is optional for Medicaid programs [31]. While the prevalence of dental insurance among medically insured adults in the San Francisco Bay area counties was 79% in the 2003 California Health Interview Survey [32], Denti-Cal--California's Medicaid fee-for-service dental program and primary public financer of dental care for more than 8 million low-income, elderly, and disabled people in California in 2007--eliminated most of its adult dental benefits in 2009 due to the state's budget deficit [33]. Though Denti-Cal coverage was in place for most of our study period, the program's dental provider reimbursement rates are among the lowest in the nation and are significantly below the fees charged by most dentists. Because of these low reimbursement rates and elimination of adult dental benefits, only 24% of California's private dentists currently accept patients with Denti-Cal, down from 40% in 2003 [33].

Though fear of dental visits is commonplace [34] and may contribute to the marked differences in percentages of patients having a dental visit versus an eye visit, a lack of healthcare provider recommendation and referral for dental care may also contribute to this finding. In turn, healthcare provider recommendation may be in part influenced by guidelines. While the American Diabetes Association guidelines strongly recommend routine retinopathy screening for patients with diabetes, there is no such recommendation for routine dental care even though periodontal disease is known to worsen glycemic control [35]. On the other hand, healthcare providers in public healthcare settings may not recommend routine dental care regardless of guidelines because accepted referrals may be limited to those with severe--and covered---dental problems such as oral abscess.

Our finding that having a dental visit varied by CKD status suggests that patients with CKD may be less likely to seek preventive dental care because of the burden of extra appointments and lifestyle modifications required for CKD management that their counterparts without CKD may not experience. Additionally, the finding may be partially attributable to out-of-pocket expenses associated with the extra appointments and medications specific to CKD management (such as phosphorus binders and vitamin D analogs). This burden would leave fewer resources for uncovered dental care expenses, but may not affect covered eye visits, for which we found no difference by CKD status. Future research should investigate the barriers to dental care specific to patients with CKD.

It is important to note that Healthy People 2010 goals for annual eye and dental examinations among people with diabetes were 76% and 71%, respectively. In 2005, 57.2% of Californians over age 18 with diabetes reported they had a dilated eye examination in the past year [36]. In 2006, 66.7% of this population reported a dental examination within the past year [37]. Our findings that only 58.3% and 14.7% of people with diabetes in our study had an eye and dental visit, respectively, over an average follow-up of > 4 years underscores the magnitude of barriers to standard of care in the public healthcare setting.

Our study is not without limitations. First, patients may have sought dental care outside of the Network. However, because of the low acceptance of private dentists of Medicaid and Medicare, we expect this was an uncommon occurrence. Also, we would not expect out-of-network care to differ by CKD status. Second, we did not have information on dentate status. Edentulous patients may have less need for dental services and are not at risk for periodontal disease. Finally, we do not have information if visits were for routine, preventive care or for problems requiring urgent attention. Given the barriers to dental care in the public healthcare setting, the rate of truly preventive dental care is likely considerably lower.

## Conclusion

In conclusion, the percentage of patients with dental care within a public healthcare setting is strikingly low overall, but particularly among those with CKD. Given the emerging association between oral health and CKD, addressing factors that impede dental access may be important for reducing the disparate burden of CKD in this population.

## Competing interests

The authors declare that they have no competing interests.

## Authors' contributions

VG participated in study design and statistical analysis and interpretation of the data, and drafted the manuscript. LCP, DST and NRP acquired the data. LCP cleaned the data and participated in statistical analysis and interpretation of the data. LCP, DST and NRP participated in study design and critical revision of the manuscript. All authors have read and approved the final manuscript.

## Pre-publication history

The pre-publication history for this paper can be accessed here:

http://www.biomedcentral.com/1471-2369/13/16/prepub
